# Diversity of Organic Acid–Producing Filamentous Fungi Isolated From Agricultural Soils of North Gondar, Ethiopia

**DOI:** 10.1155/sci5/9186819

**Published:** 2025-10-23

**Authors:** Kidist Alemayehu, Tamene Milkessa Jiru, Nega Berhane

**Affiliations:** ^1^Department of Biology, College of Natural and Computational Sciences, University of Gondar, P.O. Box 196, Gondar, Ethiopia; ^2^Department of Environmental and Industrial Biotechnology, Institute of Biotechnology, University of Gondar, P.O. Box 196, Gondar, Ethiopia; ^3^Department of Medical Biotechnology, Institute of Biotechnology, University of Gondar, P.O. Box 196, Gondar, Ethiopia

**Keywords:** agricultural soil, diversity, filamentous fungi, North Gondar, organic acid

## Abstract

**Introduction:**

Filamentous fungi are crucial for the production of commercial enzymes, organic acids, antibiotics, and many other organic compounds. Citric, acetic, and gluconic acids are among the organic acids that are produced from fungi and have many functions. They are mostly used as a chemical reagent, fungicide, herbicide, microbicide, pH adjuster, counterirritant, and solvent in a variety of industries, including food, agriculture, cleaning, and cosmetics.

**Objectives:**

This study aimed to study the diversity of selected organic acids (citric, acetic, and gluconic acids) produced by filamentous fungi isolated from the agricultural soils of North Gondar, Ethiopia.

**Methods:**

In this study, a total of 36 soil samples were randomly collected from agricultural fields at different locations in North Gondar, Ethiopia. The isolates were identified to the genus and species level based on morphological study and sequencing methods (ITS1-5·8S-ITS2 region). Their distribution was evaluated with respect to different agroecologies (climate conditions). The organic acid production capacity of the isolates was evaluated.

**Results:**

Based on the results of morphological characteristics and ITS1-5·8S-ITS2 region gene sequencing, 12 filamentous fungi were obtained. All 12 filamentous fungal isolates had one common ancestor and belonged to *Aspergillus terreus* (KIA, KIC, KID, and KIG), *Aspergillus nidulans* (KIB), *Aspergillus niger* (KIF), *Penicillium chrysogenum* (KIH), *Penicillium brevicompactum* (KII), *Talaromyces pinophilus* (KIJ), *Penicillium kongii* (KIK), *Penicillium paraherquei* (KIL), and *Talaromyces* sp. (KIM). Among these, three of them, namely, KIA, KIH, and KIF, were found to be the best producers of acetic acid, citric acid, and gluconic acid, respectively.

**Conclusion:**

Organic acid–producing filamentous fungi could be isolated from varied agroecologies.

## 1. Introduction

Microorganisms have played a crucial role in human history. From ancient civilizations, traditional practices such as fermentation have utilized microorganisms for the production of sour drinks, bread, and vinegar. Today, fungi are employed in the production of a diverse array of economically significant products, ranging from food items to biofuels and pharmaceuticals. Recent advances in our understanding of the biology, physiology, and organic chemistry of microorganisms, especially fungi, have contributed to the employment of fungi for the preparation of varied, economically valuable agricultural and industrial products [1].

Filamentous fungi are indeed a diverse and ecologically significant group of microorganisms that play essential roles in various ecosystems. They efficiently colonize substrates and decompose organic matter, contributing to nutrient cycling in their environments.

Beyond their ecological contributions, filamentous fungi have become invaluable in biotechnology and industrial applications. Their ability to produce a wide array of metabolites, including enzymes, pigments, organic acids, and secondary metabolites, has made them key players in the development of sustainable and cost-effective bioprocesses. For instance, certain filamentous fungi are known for their high yields of organic acids such as citric acid, lactic acid, and succinic acid [2]. These compounds have significant industrial applications, ranging from food preservation and flavoring to the production of biodegradable plastics and pharmaceuticals.

Organic acids are chemical compounds widely distributed in nature as normal constituents of plant or animal tissues. Organic acids constitute a key group among the building-block chemicals that can be produced by microbial processes. Organic acids have been used for many years in the food, chemical, agricultural, and pharmaceutical industries. They are used as food additives, medicative and cosmetic excipients, chemical reagents, fungicides, herbicides, microbicides, pH adjusters, medicine, etc. The chemical industries use organic acids as basic compounds for a wide variety of polymers and solvent production processes. Organic acids are used as solvents in the food, cleaning, and cosmetic industries. Organic acids act as acidifiers, which improve sensorial characteristics of beverages, contributing to color, aroma, and taste [3].

The primary types of organic acids produced by microbial activity include fumaric, propionic, lactic, itaconic, gluconic, lactobionic, citric, succinic, and acetic acids. These acids make up a developing chemical industry that produces a variety of bio-based goods [4].

Certain fungi are naturally able to produce large amounts of different organic acids. Among these, various fungi belonging to the *Aspergillus* genera can produce fumaric, itaconic, malic, citric, and gluconic acids, among others [5].

Citric acid is a weak organic acid with a molecular formula of C_6_H_8_O_7_. Filamentous fungal species, namely, *Aspergillus niger*, are efficient citric acid producers. Many other species of *Aspergillus* and varieties of yeasts, such as *Candida catenulata*, *Candida guilliermondii*, *Yarrowia lipolytica*, and *Candida tropicalis,* are also used in the production of citric acid [6].

Gluconic acid is a delicate organic acid derived from aldohexose by a straightforward oxidation reaction. Microbial production of gluconic acid is the most well-liked technique, and it dates back many decades. The foremost studied and widely used fermentation method involves its production using *A. niger* [7]. Gluconic acid and its derivatives, the principal being sodium gluconate, have wide applications in the food and pharmaceutical business.

Acetic acid (vinegar) is one of the most significant carboxylic acids. Aerobic fermentation of ethanol is the main process used to produce acetic acid. Acetic acid is a vital metabolic intermediate in biological systems and is found naturally in bodily fluids and plant juices.

Studying filamentous fungal diversity enables us to discover different kinds of metabolites, including organic acids. A number of higher fungi are involved in organic acid production [8–11]. To the best of our knowledge, the diversity of filamentous fungi that produce organic acid and that are isolated from agricultural soil of the North Gondar Zone is not studied. Studies undertaken on the diversity of filamentous fungi that produce organic acid at a global level are scanty. Therefore, this study aimed to study the diversity of selected organic acids (citric, acetic, and gluconic acids) producing filamentous fungi isolated from agricultural soils of North Gondar, Ethiopia. Potential organic acid–producing filamentous fungi were isolated, screened, and characterized.

## 2. Materials and Methods

### 2.1. Description of the Study Area

**Figure 1:**
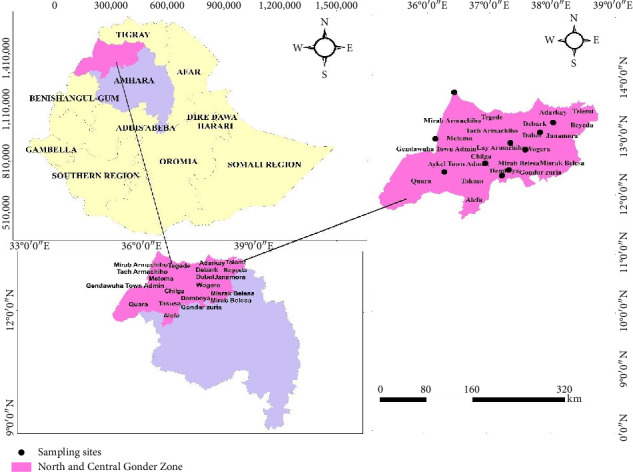
Map of study area (GIS map by Tamene Milkessa).

The study was conducted at the University of Gondar, Gondar, Ethiopia, by collecting soil samples from different parts of the North Gondar Zone, Amhara region, Ethiopia. Gondar is located 727 km from Addis Ababa (the capital city of Ethiopia) in the Northwest of Ethiopia. The study area has a dega (high land) climate with a latitude and longitude of 12°58′N 36°12′E; kola (lowland) (13°08′N 37°54′E); and weynadega (midland) (12°N 36′N 37° 28′). The altitudes of kola (lowland), weynadega (midland), and dega (highland) are 1500 m, 1500–2500 m, and > 2500 m above sea level, respectively [12]. However, the temperature ranges of the three agroecologies, i.e., dega, weynadega, and kola, include 17°C–21°C, 22°C–39°C, and > 39°C, respectively. A map of the study area is depicted in Figure 1.

### 2.2. Sample Collection

For this study, 36 randomly selected soil samples were obtained from 12 agricultural soils in the North Gondar Zone. Soil samples were collected after 10–15-cm-deep holes were dug. These samples were collected using properly labeled plastic bags in a sterile icebox. All of the collected samples were brought to the microbiology laboratory of the University of Gondar's Department of Biology and kept at 4°C for further use. Filamentous fungi were isolated from these soil samples.

### 2.3. Study Design and Period

A laboratory-based experimental study design was employed to conduct this research work from January 2023 to December 2024.

### 2.4. Isolation of Filamentous Fungi

Soil samples were diluted using known concentrations of sterile water and then plated onto potato dextrose agar (PDA) plates (g/L: potato infusion 200, dextrose 20, agar 15) and incubated. About 0.05 mg/mL of chloramphenicol was added to inhibit bacterial growth. Serial dilutions were used to prepare soil samples for analysis. About 1 gm of soil was serially diluted in 9 mL of sterile distilled water. Then, 0.1 mL of the dilution was plated onto PDA medium and incubated at 30°C for 7 days, and observations were made daily to determine the presence of discrete colonies of filamentous fungi. The fungal colonies that appeared on plates were counted as a colony-forming unit (CFU/mL) (CFU/mL = no. of colonies × dilution factor/volume of culture plate). Pure cultures of isolates were obtained by repeated subculture on PDA [13, 14].

The soil moisture content (MC) for each of the replicate samples was estimated using the following equation:

(1) $$\begin{array}{c} \%\text{ MC}=\frac{\text{Weight of moist soil }\left(M\right)-\text{Weight of dry soil }\left(D\right)}{\text{Weight of dry soil }\left(D\right)}. \end{array}$$

The pH of the soil sample was estimated using a pH meter.

### 2.5. Screening for Organic Acid Production

The production of different organic acids was checked using the Czapek–Dox broth medium as an acid indicator. The fungi were grown for 7 days in order to determine whether the medium took on a yellow color, which is a sign that acid was being produced. Additionally, to quantitatively screen the isolates for acid production, 1 mL of a 5-day-old spore suspension was inoculated in sterile liquid basal medium containing (g/L) sucrose 30, sodium nitrate 3, magnesium sulfate 0.5, potassium chloride 0.5, potassium phosphate dibasic 1.0, and ferrous sulfate 0.01. After that, the pH was adjusted to 5.0. Fermentation was carried out in 250-mL Erlenmeyer flasks with 100 mL of fermentation media at 150 rpm in a shaker incubator at room temperature (25°C). The fungal isolates that proved positive were subjected to further analysis using the titration [15, 16].

### 2.6. Molecular Identification

#### 2.6.1. Culture Preparation

For the initial step, about 50 mL of potato dextrose broth was prepared, and 10 mL of a previously grown culture was used as the inoculum. The mixture was then incubated for 72 h at 30°C. Fungal mycelial mass was recovered from the growing medium using filters with 0.45-mm pore sizes, and they were then washed with sterile saline.

#### 2.6.2. DNA Extraction

The filamentous fungi that were selected and evaluated for their ability to produce various organic acids were initially cultured for 72 h at 30°C on PDA medium. Following incubation, 500 μL of extraction buffers (100 mM Tris–HCl [pH 8.0], 20 mM EDTA [pH 8.0], 1.4 M NaCl, 2% CTAB, and 0.2% 2-mercaptoethanol) was used to grind the fungal mycelial mass off the culture plate in a sterile mortar and pestle. The mixture was then centrifuged for 10 min at 14,000 rpm. Following the manufacturer's instructions, the GenElute Plant Genomic DNA Purification Kit (Sigma-Aldrich) was used to extract genomic DNA from pellets of isolated filamentous fungi. The DNA served as a template for amplification using the polymerase chain reaction (PCR) and was kept at −20°C.

#### 2.6.3. PCR Amplification

PCRs of the internal transcribed spacer (ITS1-5·8S-ITS2) region of filamentous fungi were amplified using the universal fungal primer ITS1 (forward primer) 5′-TCCGTAGGTGAACCTGCGG-3′ and ITS4 (reverse primer) 5′-TCCTCCGCTTATTGA TATGC-3′ [17, 18]. PCRs were performed in 50 μL of PCR master mix solution (Promega, United States). The following amplification steps were followed: initial denaturation at 95°C for 5 min followed by 40 cycles consisting of denaturation at 95°C for 30 s, an annealing step at 50°C for 1 min, and extension at 72°C for 1 min, respectively, and a final extension at 72°C for 5 min. The PCR products were separated using electrophoresis on 1% agarose gel containing 0.7 mg/mL of ethidium bromide and seen under UV light in a gel documentation cabinet. The approximate size of amplicons was determined using standard molecular markers (GeNetbio 100-bp DNA ladder).

#### 2.6.4. Sequence Analysis

PCR-amplified products (bands) of filamentous fungal isolates were eluted and sent to Macrogen Company, the Netherlands, and were sequenced with the incorporation of dideoxynucleotides (ddNTPs) in the reaction mixture using forward and reverse directions using the ITS1 primer (TCCGTAGGTGAACCTGCGG) and the ITS4 primer (TCCTCCGCTTATTGATATGC), respectively [18].

#### 2.6.5. Phylogenetic Tree Analysis

The ITS1-5.8S-ITS2 sequences obtained were further analyzed using the National Center for Biotechnology Information's (https://www.ncbi.nlm.nih.gov/BLAST) BLASTN search alignment tool to find similar sequences in the GenBank database. For phylogenetic analysis, the available gene sequence data of related organisms were retrieved in FASTA format and aligned using Clustal-W [19, 20]. The phylogenetic tree was constructed using the neighbor-joining (NJ) technique, and distances were computed with the help of the maximum composite likelihood method. The branching patterns were evaluated using 1000 bootstrap replicates. Lastly, the sequencing finding was deposited in the NCBI database, and accession numbers were obtained for each isolate.

#### 2.6.6. Estimation of Total Titratable Acidity in Selected Fungi

The total acid content of the culture filtrate was measured against 10 mL of fermentation broth against 0.1 N NaOH (alkaline standard) using phenolphthalein as an indicator, and the intensity of acid production was determined by molarity. A few drops of phenolphthalein indicator were added to 1 mL of culture filtrate in the flask and then filtered with 0.1 N NaOH. The point at which the filtrate changes from being colorless to pink was determined to be the termination point. Identification procedures were carried out on the isolates with the highest acid productivity [16].

## 3. Results

### 3.1. Screening of Organic Acid–Producing Bacteria

In this study, 36 soil samples were collected from 12 agricultural sites in the North Gondar Zone of the Amhara regional state, Ethiopia. From these samples, approximately 30 fungal isolates were successfully obtained. These isolates were then evaluated for their capacity to produce organic acids, specifically acetic acid, citric acid, and gluconic acid, using the Czapek–Dox broth medium as a medium for acid detection.

The primary screening process identified 12 fungal isolates that demonstrated acidophilic activity, as indicated by a color change in the medium from a watery appearance to yellow. This visual change suggests the production of acids by the fungi. To further validate these findings, total titratable acidity measurements were conducted on the selected fungal isolates, which supported the initial results obtained from the Czapek–Dox broth growth assays (data not shown here).

### 3.2. MC, pH, and Microbial Load

The moisture levels in the soil samples ranged from 10.0% to 12.2%. This suggests that the soils have a relatively moderate moisture level, which could impact the growth and activity of fungi. The pH values of the soil ranged from 4.7 to 6.0. This indicates that the soils are slightly acidic to neutral, which is a common pH range for many types of fungi. The pH can greatly influence the microbial community structure in soil. The microbial load, measured in CFU/mL, ranged from 2.6 to 8.71 × 10^4^ CFU/mL. This range indicates a significant presence of filamentous fungi in the samples, which is important for soil health and nutrient cycling. The detailed descriptions are depicted in Table 1.

### 3.3. Morphological Characterization

In fungal systematics, morphological characterization serves as a foundational step before molecular identification. This approach remains crucial, as it allows for the preliminary classification and identification of fungal isolates based on observable characteristics. In this study, fungal isolates that had already been screened were subjected to a detailed morphological analysis. The results regarding the colony diameter of rot, colony character, colony surface color, and color zonation of different organic acid–producing fungi are indicated in Table 2.

### 3.4. Molecular Characterization of Filamentous Fungi

**Figure 2:**
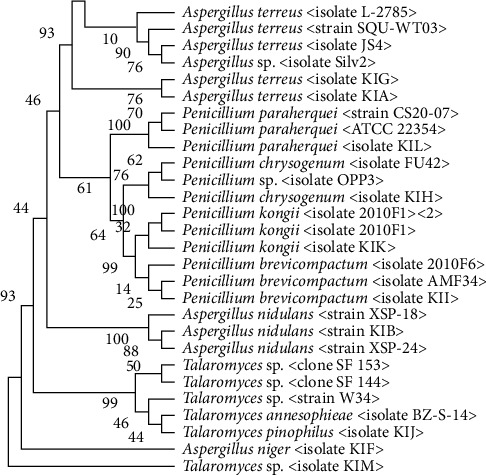
Phylogenetic tree of the ITS1-5.8S-ITS2 rDNA region gene sequence of fungal isolates in this study with related fungal species of the NCBI database. The neighbor-joining (NJ) method of MEGA 11 software was used to construct the tree, and the evolutionary distances were computed with the maximum composite likelihood method. The branching patterns were checked using a 1000-bootstrap replicate.

Following morphological characterization, identification, and screening for the production of organic acids, filamentous fungi were further identified through ITS1-5.8S-ITS2 sequence analysis. The sequences derived from this study and those of closely related sequences from the NCBI database (GenBank) are shown in Figure 2. The majority of the isolated fungi shared 90%–99% of their characteristics with the related filamentous fungi listed in GenBank. The isolated fungi were deposited in the GenBank database under accession numbers given in parentheses. The isolated organisms are obtained as follows: KIA (*Aspergillus terreus* 1, Acc. No.: OR038984), KIB (*Aspergillus nidulans*, Acc. No.: OR038985), KIC (*A. terreus* 2, Acc. No.: OR038986), KID (*A. terreus* 3, Acc. No.: OR038987), KIF (*A. niger*, Acc. No.: OR038988), KIG (*A. terreus* 4, Acc. No.: OR038989), KIH (*Penicillium chrysogenum*, Acc. No.: OR038990), KII (*P. chrysogenum*, Acc. No.: OR038991), KIJ (*Trichoderma pinophilus*, Acc. No.: OR038992), KIK (*Penicillium kongii*, Acc. No.: OR038993), KIL (*Penicillium paraherquei*, Acc. No.: OR038994), and KIM (*Talaromyces* sp., Acc. No.: OR038995).

### 3.5. Organic Acid Production Using Selected Filamentous Fungi

The study on acidophilic fungi indicated that several species were present across various study sites, displaying varying capabilities for acid production. Specifically, the fungi *A. terreus* (Acc. No.: OR038984), *P. chrysogenum* (Acc. No.: OR038990), and *A. niger* (Acc. No.: OR038988) were identified as producers of acetic, citric, and gluconic acids in their culture filtrate.

The titration analysis provided quantitative data regarding the acid production, revealing that the maximum concentrations of acids were as follows: *A. terreus* produced up to 72.50 g/L of acetic acid, *P. chrysogenum* yielded 65.00 g/L of citric acid, and *A. niger* generated 66.00 g/L of gluconic acid (as summarized in Table 3). These findings highlight the significant metabolic capabilities of these fungi in acidic environments, which may have implications for their application in various biotechnological processes.

## 4. Discussion

Organic acids have long been used in the food, chemical, agricultural, and pharmaceutical industries. The production of various solvents and polymers is one of the numerous chemical businesses that can benefit from these organic acids as building blocks. The elements of carbon, hydrogen, and oxygen that are involved cause them to differ. The primary types of organic acids produced by microbial activity include fumaric, propionic, lactic, itaconic, gluconic, lactobionic, citric, succinic, and acetic acids. Many bio-based chemicals are generated from these acids, which make up a developing chemical segment [4].

In this study, a total of 36 agricultural soil samples were collected from various locations in North Gondar. From these samples, approximately 30 fungal isolates were identified. These isolates were screened for their ability to produce organic acids, specifically acetic, citric, and gluconic acids. The screening process yielded 12 promising organic acid–producing filamentous fungi, which have been designated as KIA, KIB, KIC, KID, KIF, KIG, KIH, KII, KIJ, KIK, KIL, and KIM.

Additionally, the study assessed the pH and MC of the soil samples from which these 12 isolates were obtained. The MC of the soil samples ranged from 10.0% to 2.2%, while the pH levels varied between 4.7 and 6.0. The microbial load of the selected organic acid–producing fungal isolates was found to range from 2.6 to 8.71 × 10^4^ CFU/mL.

Liaud et al. [10] successfully isolated and screened 66 strains of filamentous fungi for organic acid production, indicating a significant interest in harnessing these microorganisms for their biochemical potential. Dezam et al. [11] focused on endophytic strains from mangrove ecosystems in São Paulo, Brazil, discovering 35 organic acid–producing strains. This work highlights the ecological role of fungi in mangrove habitats and their potential utility. Mukunda et al. [21] explored unexplored soils in the Western Ghats, leading to the isolation of over 200 fungal strains. Their work on screening for hydrolytic enzymes, such as amylase and cellulase, emphasizes the importance of these fungi in soil ecosystems and possible applications in waste degradation and bioprocessing. They characterized a total of 167 isolates, identifying representative species predominantly within the genera *Aspergillus*, *Penicillium*, *Trichoderma*, and *Cladosporium*. Khan and Gupta [22] specifically targeted acidophilic fungi from a mining area in Chhattisgarh, isolating 29 strains. Their qualitative and quantitative analysis showed that some isolates from *Aspergillus* and *Penicillium* were particularly effective at acid production.

In this study, 12 filamentous fungal isolates were initially characterized morphologically, followed by confirmation through molecular identification methods. The morphological characteristics, in conjunction with ITS1-5.8S-ITS2 gene sequencing, revealed that most of the isolated fungi shared 90%–99% similarity with related filamentous fungi documented in GenBank. Analysis of these isolates indicated a common ancestor, with classifications including *A. terreus* (isolates KIA, KIC, KID, and KIG), *A. nidulans* (isolate KIB), *A. niger* (isolate KIF), *P. chrysogenum* (isolate KIH), *Penicillium brevicompactum* (isolate KII), *Trichoderma pinophilus* (isolate KIJ), *P. kongii* (isolate KIK), *P. paraherquei* (isolate KIL), and an unidentified *Talaromyces* sp. (isolate KIM). This study highlighted that *Aspergillus* and *Penicilliu*m are the predominant acidophilic fungi in the North Gondar Zone of the Amhara region in Ethiopia. Diverse acidophilic fungi exhibiting varying capacities for acid generation were observed across all study sites. According to Klich [8], representatives of *Aspergillus* and *Penicillium* can thrive in various environmental conditions, including mining environments. Numerous species within these genera are prevalent in nature, with some known for their production of organic acids, further reinforcing their ecological significance and versatility.

The exploration of fungi, particularly those within the genus *Aspergillus*, for the production of organic acids is gaining significant traction due to their commercial applications and market potential. Ferreira et al. [23] highlight the utility of *Aspergillus* species in this context, showcasing their ability to produce valuable organic acids. This is echoed by Rathod et al. [24], who note that widely distributed fungi, such as *Penicillium*, *Aspergillus*, *Chaetomium*, and *Trichoderma*, are commonly found in various environments, including mining regions. Recent findings indicate the presence of several fungal species, such as *A. niger*, *A. terreus*, *Aspergillus flavus*, and various *Penicillium* species, supporting the ongoing research in this area [9, 25]. The organic acids produced by these microorganisms during fermentation processes play a crucial role in metal leaching, as noted by Jain and Sharma [26]. Vezzola et al. [27] also studied another species of *Aspergillus*, *Aspergillus tubingensis*. This strain was able to produce organic acid, which is useful for metal extraction (bioleaching). This finding underscores the significance of fungal fermentation not only for organic acid production but also for environmental remediation and resource recovery. Yang [28] further contributes to this field by examining the potential of specific *Aspergillus* strains, such as *Aspergillus carbonarius* ITEM5010 and *Aspergillus saccharolyticus* IBT28231, as industrial cell factories for succinic acid production. While *A. carbonarius* is noted for its production of citric and gluconic acids under varied pH conditions, it does not produce succinic acid. However, *A. saccharolyticus* demonstrates a capacity for producing both malic and succinic acids in buffered pH conditions. This suggests that different strains of *Aspergillus* may be optimized for specific organic acid productions, highlighting the importance of strain selection in industrial applications. Studies also suggest that *Talaromyces* species, such as *Talaromyces pinophilus*, produce the highest amount of acetic, formic, and lactic acids in the medium supplemented with Ca_3_(PO_4_)_2_, citric and oxalic acids in the medium supplemented with AlPO_4_, and tartaric and malic acids in the medium supplemented with FePO_4_ [29]. Zúñiga-Silgado et al. [30] isolated three *Trichoderma* and one *Aspergillus* organic acid–secreting species from soil. These fungi were found to be efficient phosphate solubilizers.

Overall, these studies illustrate the richness of fungal biodiversity in different ecological niches and their potential for industrial applications, including organic acid production, enzyme production, and even bioremediation. The findings could inspire further research into the biotechnological exploitation of filamentous fungi for sustainable practices.

## 5. Conclusion

The study highlights the successful isolation of 12 potential organic acid–producing filamentous fungi from agricultural soils in the North Gondar Zone. These findings underscore the rich biodiversity of fungal species in the region, particularly the presence of genera such as *Aspergillus* and *Penicillium*. These fungi are known for their ability to produce significant amounts of organic acids, including acetic, gluconic, and citric acids, which are essential for various applications in agriculture and the food industry.

The identified fungi could have practical implications, such as enhancing soil fertility through organic acid production, which can help in nutrient solubilization and improving plant growth. The study opens avenues for further research into the genetic and metabolic pathways of these fungi, with the potential for biotechnological applications in sustainable agriculture. The results suggest that the agricultural soils of the North Gondar Zone may serve as a valuable resource for discovering effective organic acid–producing fungi that could contribute to agricultural productivity and ecological balance.

## Figures and Tables

**Table 1:** Sample collection locations, moisture content, pH, and microbial load of organic acid–producing filamentous fungi from North Gondar Zone.

Fungal isolate	Sample collection site	Altitude (m)	Latitude and longitude	Moisture content	Soil pH	CFU/mL (*x* × 10^4^)
KIA	Metema	685	12°57′16.06″N, 36°9′26.12″E	10.1	5.0	8.71
KIB	Quara	1713	12° 18′0″N, 36°13′0″E	10.6	5.3	7.7
KIC	Metema	685	12°57′16.06″N, 36°9′26.12″E	10.0	5.1	5.6
KID	Debark	2850	13°9′22″N, 37° 53′53″E	11.2	4.9	7.4
KIF	Soroka	849	64°31′31″N, 34°45′56″E	10.8	6.0	6.6
KIG	Gondar	2133	12°35′59.99″N, 37°27′59.99″E	11.8	5.4	5.0
KIH	Gondar	2133	12°32′N, 37°26′E	11.5	5.1	5.1
KII	Tikil Dingay	2243.65	12° 59′ 3″N, 37°2′39″E	11.6	4.7	4.9
KIJ	Kola Diba	2146	12°25′21.70″N, 37°19′26.18″E	11.3	5.4	5.2
KIK	Amba Giorgis	2960.71	12°46′6.36″N, 37° 37′31.28″E	12.0	4.8	4.8
KIL	Chilga	2146	12° 33′ 0″N, 37°4′0″E	12.2	5.0	4.6
KIM	Chilga	2146	12° 33′ 0″N, 37°4′0″E	12.1	5.2	2.6

**Table 2:** Colony morphological characteristics of fungal isolates cultured on PDA after 7 days of incubation at 30°C.

Fungal species	Colony character	Surface color	Color zonation
KIA	Velvety thick	Creamish thick	Yellow soluble pigments on the reverse
KIB	Conidia	Dark green/granular colony on the top	Hyaline, pale olive on the reverse
KIC	Brush arrangement of phialospores	Pinkish	Pale yellow on the reverse
KID	Brush arrangement of phialospores	Pinkish cinnamon to deeper with age	Yellow on the reverse
KIF	Wrinkled, globular, and warty conidia	Turning black color on the top	Light yellow color on the reverse
KIG	Brush arrangement of phialospores	Pinkish	Light yellow on the reverse
KIH	Conidiophores	Grayish olive	Pale to pale gray on the reverse
KII	Conidiophores	Yellow–brown	Yellowish on the reverse
KIJ	Conidiophores	Simple green tightly	White at margins on the reverse
KIK	Conidium	Gray–green	Yellowish on the reverse
KIL	Branching of individual hyphae	Green	Yellowish
KIM	Conidiophores	Yellowish green to vivid green	Pink at centers

**Table 3:** Quantity of organic acids in culture filtrates of *A. terreus* (OR038984) and *P. chrysogenum* (OR038990) following 7 days of growth.

Fungal isolates	Organic acids (g/L)	Organic acids (g/L)	Organic acids (g/L)
	Acetic acid	Citric acid	Gluconic acid
KIA (*A. terreus* 1)	72.50	7.5	23.2
KIH (*P. chrysogenum*)	20.11	6.9	66.32
KIF (*A. niger*)	30.10	65	11.24

## Data Availability

The data used to support the findings of this study are included within the article.
